# Acute pulmonary edema after subarachnoid hemorrhage: risk factors and comorbidities—an analysis of a nationwide database from the United States

**DOI:** 10.1186/s40560-025-00796-w

**Published:** 2025-05-13

**Authors:** Alejandro Pando, Anil Kumar Tenneli, T. Pradeep, Priyanka Augustine, Balamurali Krishna, Jeffrey Pradeep Raj

**Affiliations:** 1 Department of Neurological Surgery, Rutgers New Jersey Medical School, Rutgers Robert Wood Johnson Medical School, Newark, NJ USA; 2 Department of Pediatrics, Indira Gandhi Institute of Child Health, Bangalore, KA India; 3 Department of Pharmacology, Karpagam Faculty of Medical Sciences and Research, Coimbatore, TN India; 4 Internal Medicine Trainee-Department of Respiratory Medicine, Sheffield Teaching Hospitals, Sheffield, UK; 5 Department of Respiratory Medicine, KLES Dr Prabhakar Kore Hospital & Medical Research Centre, Belagavi, KA India; 6 Division of Clinical Pharmacology, Department of Pharmacology, Kasturba Medical College Manipal, Manipal Academy of Higher Education, Manipal, KA India

**Keywords:** Subarachnoid hemorrhage, Pulmonary edema, Neurogenic pulmonary edema, National inpatient sample, Risk factors, Comorbidity, Hospital mortality, Length of stay, Health care costs

## Abstract

**Background:**

Acute pulmonary edema (APE) is a rare complication of subarachnoid hemorrhage (SAH) that is associated with increased morbidity and poor clinical outcomes. There is limited literature addressing the incidence and risk factors of this complication, highlighting the need for further investigation as undertaken in the present study.

**Methods:**

The 2016 to 2021 National Inpatient Sample (NIS) was used to identify adult inpatients with a primary diagnosis of non-traumatic SAH. Univariate and multivariable analyses adjusting for patient demographics, and comorbidity status, were used to characterize statistical associations with APE.

**Results:**

A total of 42,141 patients were identified as having SAH from 2016 to 2021. Of these patients, 960 patients (2.3%) were found to have APE. APE was associated with increased length of stay (20.0 ± 18.9 days vs. 11.6 ± 14.3, p < 0.001), increased total costs ($503,671.3 ± 647,729.9 vs. $238,724.6 ± 328,062.1, p < 0.001), increased number of days from admission to first procedure (3.5 ± 7.3 vs. 1.9 ± 4.9, p < 0.001), increased Elixhauser comorbidity index ≥ 3 (77.5% vs. 66.0%, p < 0.001), and increased mortality (40.2% vs. 22.5%, p < 0.001). After controlling for confounding factors, independent risk factors for APE in patients with non-traumatic SAH included: Coagulopathies (adjusted Odds Ratio [aOR]: 1.57, 95% confidence interval [CI] 1.31–1.89, p < 0.001), Fluid and Electrolyte Disorders (aOR: 2.54, CI 2.13–3.03, p < 0.001), Liver Disease (aOR: 1.37, CI 1.07–1.76, p = 0.013), Obesity (aOR: 1.47, CI 1.19–1.81, p = 0.003), Pulmonary Circulatory Disorder (aOR: 1.72, CI 1.31–2.26, p = 0.001), and Weight Loss (aOR: 1.67, CI 1.36–2.04, p < 0.001).

**Conclusion:**

APE after SAH is associated with increased complicated hospital course. Neurosurgeons and Neurocritical care medical professionals should be aware of the comorbidities and factors associated with increased APE after SAH to improve patient outcomes.

**Supplementary Information:**

The online version contains supplementary material available at 10.1186/s40560-025-00796-w.

## Background

Acute pulmonary edema (APE) is a critical and potentially life-threatening condition characterized by the rapid accumulation of fluid in the alveolar spaces of the lungs, leading to impaired gas exchange and respiratory failure. This may be due to cardiogenic causes such as acute left ventricular dysfunction or non-cardiogenic mechanisms, including infections or central nervous system insults [1]. APE is associated with significant morbidity, prolonged hospitalization, increased healthcare costs, and high in-hospital mortality, particularly among critically ill patients [1, 2]. Despite its clinical importance, the epidemiology and risk factors for APE remain underreported, especially in neurologically compromised populations.

Among neurologic conditions, subarachnoid hemorrhage (SAH) represents a recognized but relatively less studied precipitant of APE. SAH, whether traumatic or non-traumatic, poses a life-threatening risk due to blood accumulation between the arachnoid and pia mater, elevating intracranial pressure. The most common cause is non-traumatic due to rupture of an aneurysm [3, 4]. Pulmonary complications are reported in up to 50% of patients with SAH, of which APE is a key contributor to respiratory distress and adverse outcomes [5]. The pathogenesis of APE in this context is likely multifactorial—ranging from sympathetic hyperactivation and inflammatory responses to volume overload or pre-existing cardiopulmonary comorbidities [1, 5]. A distinct subtype, neurogenic pulmonary edema (NPE), has been described in SAH and other acute brain injuries, but distinguishing it from other causes of APE is challenging in routine clinical settings [6].

Given the complex and overlapping mechanisms behind APE in SAH, and the lack of large-scale epidemiological studies characterizing this phenomenon, there is a pressing need to delineate its burden and risk factors more clearly. This study aims to identify the incidence and comorbidity patterns associated with APE among adult patients with non-traumatic SAH using data from a large, nationally representative inpatient database.

## Methods

### Ethics

Since the study was retrospective and utilized publicly available data, it did not require review by the institutional review board. The research adhered to the principles outlined in the Declaration of Helsinki (Fortaleza, Brazil, 2013), as well as the Good Clinical Practice guidelines (E6-R2) established by the International Council for Harmonization of Technical Requirements for Pharmaceuticals for Human Use (ICH) (Maryland, USA, 2018).

### Study design and setting

This study involved a retrospective analysis of deidentified patient data sourced from the National Inpatient Sample (NIS), one of the nation’s largest databases containing inpatient healthcare information. The NIS forms a vital component of the Healthcare Cost and Utilization Project (HCUP), supported by the Agency for Healthcare Research and Quality (AHRQ). It is important to note that neither HCUP nor AHRQ are accountable for the interpretations or conclusions drawn from this analysis.

### Eligibility criteria

The database from 2016 to 2021 from the NIS was utilized to identify adult inpatients (age ≥ 18 years) of any sex with a primary diagnosis of non-traumatic SAH-International Classification of Diseases, 10th Revision, (ICD-10) Clinical Modification (CM) codes I60.0–I60.9 were selected. There were no exclusions.

### Variables

The records were divided into two groups: those with APE and those without. A clinical diagnosis of APE was based on the ICD-10 code J81.0 as recorded in the NIS database entered by coders based on physician documentation of clinical and radiological findings. The exact underlying clinical/radiological findings that led to the diagnosis are not available in the database.

Data collection focused on two primary areas:

- Demographic features: Including age, sex, race, patient location, insurance status, and similar variables.
- Clinical information: Covering length of stay, expenses, smoking status, mortality, Elixhauser comorbidity index, and select comorbidities identified using ICD-10 codes (Supplementary Table 1) included in the Elixhauser comorbidity software by HCUP.

### Study procedures and data sources

Participants who satisfied the eligibility criteria had their records electronically retrieved from the NIS database. A digital data extraction form, designed by the first author, was utilized to gather relevant information, which was subsequently employed for statistical analysis. We have done a similar analysis for neurogenic stress cardiomyopathy in non-traumatic SAH, which has been recently published [7].

### Mitigation of bias

To address potential sources of bias inherent to retrospective observational studies, several methodological strategies were employed. Selection bias was controlled by analyzing a large, nationally representative sample drawn from the National Inpatient Sample (NIS) database over a 6-year period, thereby enhancing population generalizability and reducing sampling error. Recall bias was inherently minimized due to the use of routinely collected real-time administrative and clinical data, rather than relying on participant self-report or retrospective interviews [8]. To mitigate bias in model construction, we adopted a causal inference framework guided by a Directed Acyclic Graph (DAG) to inform variable selection for multivariable analysis. This approach reduced the risk of collider bias and overfitting by including only those variables with theoretical and biological plausibility, independent of statistical significance in univariate analysis. Variables identified as potential sources of spurious associations—such as uncomplicated hypertension, hypothyroidism, and non-metastatic solid tumors—were excluded based on causal structure.

### Sample size estimation

A formal sample size calculation was not conducted, as all eligible participant records from a 6-year timeframe, spanning from January 1, 2016, to December 31, 2021, were incorporated.

### Data management

Patient data extraction was performed utilizing Microsoft® Excel for Microsoft 365, version 2206—Build 15330.20306, provided by Microsoft Corporation, Redmond, Washington, USA, 2022, and password protected. Subsequently, the de-identified patient data underwent analysis using the Statistical Analysis System for Windows, Version 9.4, developed by SAS Institute Inc., Cary, North Carolina, USA, 2013.

### Quantitative variables and statistical methods

Demographic characteristics and clinical data including a history of comorbidities were summarized using descriptive statistics. The normal distribution of data was assessed using Kolmogorov–Smirnov’s normality test. Continuous variables were expressed as Mean (Standard Deviation—SD), while categorical variables were presented as frequency (percentage). Between-group comparisons (APE versus no APE) were made using z-test with p-values adjusted using the Bonferroni method. The z-test was used for comparisons of continuous variables between groups owing to the large sample size, where the sampling distribution approximates normality, and the sample standard deviation reliably estimates the population standard deviation. Results were verified with Student’s t-test and yielded consistent findings.

Following univariate analysis, we conducted multivariable logistic regression to examine associations with APE. Variable selection was informed by a DAG, developed using clinical knowledge, pathophysiological plausibility, and prior literature. This approach prioritized the inclusion of variables with theoretical relevance rather than relying solely on statistical significance in univariate analysis. The final model included age, sex, and the following comorbidities: coagulopathy, congestive heart failure, fluid and electrolyte disorders, hypertension (complicated), liver disease, metastatic cancers, neurologic disorders, obesity, pulmonary circulatory disorders, and weight loss. Comorbidities with unclear causal pathways or potential to introduce collider bias—such as uncomplicated hypertension, hypothyroidism, and solid tumors without metastasis—were excluded. To maintain model parsimony and minimize the risk of overfitting, the number of covariates was limited based on the number of APE events in the dataset. We also assessed for and avoided inclusion of collinear variables, especially those with overlapping constructs within the Elixhauser comorbidity index. The theoretical justifications for variable inclusion are provided in Supplementary Table 2, and the DAG used to guide the model is presented in Supplementary Fig. 1. Statistical significance for all analyses was defined as p < 0.05.

## Results

**Fig. 1:**
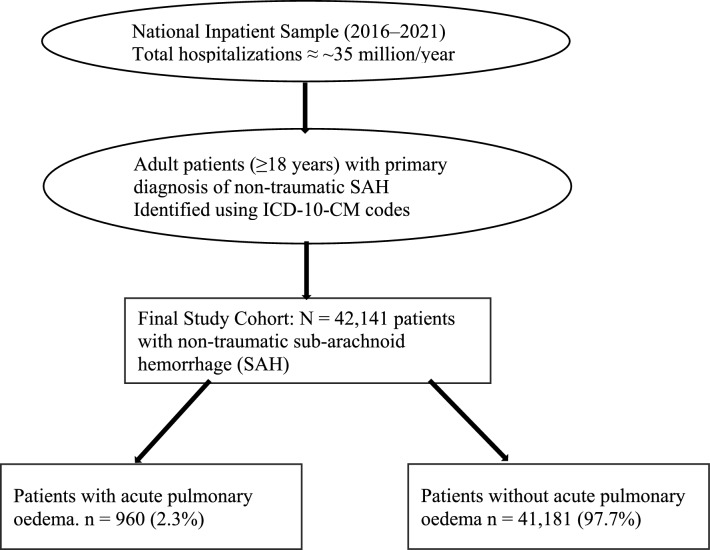
Patient selection flowchart

A total of 42,141 patients were identified with non-traumatic SAH, among whom 960 (2.3%) had APE. The patient selection flowchart is given in Fig. 1. The mean (SD) age of the entire study sample was 62.0 (16.8) years, with 23,946 out of 42,141 (56.8%) being women. The mean (SD) age of individuals with APE was 58.0 (± 16.9) years, significantly lower (p < 0.001) compared to those without APE, whose mean age was 62.1 (± 16.8) years. Similarly, 527 out of 960 (54.9%) were women, with no significant difference observed between those with and without APE. The average duration of stay in the APE group was 20.0 ± 18.9 days, significantly longer than the non-APE group (11.6 ± 14.3 days, p < 0.001), while the mean total cost was notably higher in the APE group ($503,671.3 ± 647,729.9 versus $238,724.6 ± 328,062.1, p < 0.001). Additionally, mortality rates (40.2% versus 22.5%) and Elixhauser comorbidity index ≥ 3 (77.5% vs. 66.0%) were significantly elevated in the APE group. Further details regarding demographic characteristics are provided in Table 1.

**Table 1:** Demographic characteristics

Category	Total # of patients with SAH included in the analysis	SAH patients with APE	SAH patients without APE	p value^1^
Number of admissions (N [%])	42,141 (100)	960 (2.3)	41,181 (97.7)	–
Mean age (year)	62.0 ± 16.8	58.0 ± 16.9	62.1 ± 16.8	< 0.001
Sex (N [%])				
Male	18,189 (43.2)	433 (45.1)	17,756 (43.1)	0.554
Female	23,946 (56.8)	527 (54.9)	23,419 (56.9)	0.550
N/A	6 (0.0)	0 (0)	6 (0)	
Race (N[%])				
White	25,061 (59.5)	549 (57.2)	24,512 (59.5)	0.733
Black	6568 (15.6)	144 (15)	6424 (15.6)	0.317
Hispanic	4917 (11.7)	135 (14.1)	4782 (11.6)	0.997
Asian or Pacific Islander	1890 (4.5)	43 (4.5)	1847 (4.5)	0.479
Native American	250 (0.6)	7 (0.7)	243 (0.6)	0.414
Other	1691 (4.0)	39 (4.1)	1652 (4.0)	0.473
N/A	1764 (4.2)	43 (4.5)	1721 (4.2)	0.232
Mean length of stay (days)	11.8 ± 14.5	20.0 ± 18.9	11.6 ± 14.3	< 0.001
Mean total charge	$244,756.8 ± 340,996.5	$503,671.3 ± 647,729.9	$238,724.6 ± 328,062.1	< 0.001
Mean until first procedure (days)	1.9 ± 5.0	3.5 ± 7.3	1.9 ± 4.9	< 0.001
Mortality (N [%])				
Died	9664 (22.9)	386 (40.2)	9278 (22.5)	< 0.001
Did not die	32,456 (77.0)	574 (59.8)	31,882 (77.4)	< 0.001
Primary insurance (N [%])				
Medicare	19,669 (46.7)	360 (37.5)	19,309 (46.9)	< 0.001
Medicaid	6453 (15.3)	175 (18.2)	6278 (15.2)	0.961
Private insurance	12,160 (28.9)	331 (34.5)	11,829 (28.7)	< 0.001
Self-pay	2309 (5.5)	49 (5.1)	2260 (5.5)	< 0.001
No charge	178 (0.4)	5 (0.5)	173 (0.4)	> 0.999
Other	1277 (3.0)	36 (3.75)	1241 (3.0)	0.824
N/A	95 (0.2)	4 (0.4)	91 (0.2)	0.693
Patient location (N [%])				
Counties in metro areas of 250,000–999,999 population	8647 (20.5)	171 (17.8)	8476 (20.6)	0.094
Counties in metro areas of 50,000–249,999 population	3717 (8.8)	73 (7.6)	3644 (8.8)	0.138
Micropolitan counties	3614 (8.6)	87 (9.1)	3527 (8.6)	> 0.999
“Central” counties of metro areas of ≥ 1 million population	13,285 (31.5)	311 (32.4)	12,974 (31.5)	0.560
“Fringe” counties of metro areas of ≥ 1 million population	9968 (23.7)	243 (25.3)	9725 (23.6)	0.281
Not metropolitan or micropolitan counties	2629 (6.2)	67 (7.0)	2562 (6.2)	0.316
N/A	281 (0.7)	8 (0.8)	273 (0.7)	0.475
Elixhauser comorbidity index (N [%])				
0	1837 (4.4)	16 (1.7)	1821 (4.4)	0.054
1	4859 (11.5)	60 (6.3)	4799 (11.7)	0.013
2	7538 (17.9)	140 (4.6)	7398 (18.0)	0.119
≥ 3	27,907 (66.2)	744 (77.5)	27,163 (66.0)	< 0.001
N/A	0	0	0	NA
Smoking status				
Yes	5750 (13.6)	125 (13.0)	5625 (13.7)	0.553
No	36,391 (86.4)	835 (87.0)	35,556 (86.3)	0.553

*N/A* missing data

^1^Comparisons were conducted using the z-test with p-values adjusted using the Bonferroni method

Among individuals with nontraumatic SAH, the prevailing comorbidities included hypertension (present in 29,803 out of 42,141 patients, accounting for 70.7%), followed by fluid and electrolyte imbalance (present in 20,972/42,141 patients, accounting for 49.8%), and cardiac arrhythmias (present in 11,929/42,141 patients, accounting for 28.3%). When comparing the APE group against the no APE group, those with APE had a significantly higher incidence of coagulopathy (26.5% versus 13.4%), fluid and electrolyte imbalance (75.2% versus 49.2%), liver disease (11.8% versus 5.6%), obesity (15.5% versus 11.9%), and weight loss (16.5% versus 8.9%). The APE group had a significantly lower incidence of uncomplicated hypertension (40.6% versus 53.4%), solid tumors without metastasis (1.9% versus 4.2%) and metastatic cancer (1.4% versus 3.0%). The details of the comorbidities analyzed are summarized in Table 2.

**Table 2:** Comorbidities

Category	Total # of patients with SAH included in the analysis (N [%])	SAH patients with APE (N [%])	SAH patients without APE (N [%])	p value^1^
Acquired immune deficiency syndrome (AIDS)	124 (0.3)	4 (0.4)	120 (0.3)	0.949
Alcohol abuse	83 (0.2)	2 (0.2)	81 (0.2)	0.640
Blood loss anemia	261 (0.6)	4 (0.4)	257 (0.6)	0.383
Cardiac arrhythmias	11,929 (28.3)	297 (30.9)	11,632 (28.2)	0.141
Chronic deficiency anemia	1203 (2.9)	32 (3.3)	1171 (2.8)	0.851
Chronic lung disease	6104 (14.5)	139 (14.5)	5965 (14.5)	0.835
Coagulopathy	5791 (13.7)	254 (26.5)	5537 (13.4)	< 0.001
Congestive heart failure	5985 (14.2)	178 (18.5)	5807 (14.1)	0.376
Depression	4347 (10.3)	75 (7.8)	4272 (10.4)	0.163
Diabetes mellitus, uncomplicated	3883 (9.2)	58 (6.0)	3825 (9.3)	0.150
Diabetes mellitus, complicated	5336 (12.7)	117 (12.2)	5219 (12.7)	0.847
Drug abuse	2759 (6.5)	64 (6.7)	2695 (6.5)	0.253
Fluid and electrolyte disorder	20,972 (49.8)	722 (75.2)	20,250 (49.2)	< 0.001
Hypertension, uncomplicated	22,362 (53.1)	390 (40.6)	21,972 (53.4)	< 0.001
Hypertension, complicated	7441 (17.7)	160 (16.7)	7281 (17.7)	0.340
Hypothyroidism	4589 (10.9)	70 (7.3)	4519 (11.0)	0.100
Liver disease	2415 (5.7)	113 (11.8)	2302 (5.6)	< 0.001
Neurologic disorders	7974 (18.9)	220 (22.9)	7754 (18.8)	0.478
Obesity	5058 (12.0)	149 (15.5)	4909 (11.9)	0.030
Solid tumors without metastasis	1728 (4.1)	18 (1.9)	1710 (4.2)	0.017
Metastatic cancer	1242 (2.9)	13 (1.4)	1229 (3.0)	0.018
Lymphoma	203 (0.5)	9 (0.9)	194 (0.5)	0.911
Paralysis	9424 (22.4)	200 (20.8)	9224 (22.4)	> 0.999
Peptic ulcer disease excluding bleeding	201 (0.5)	1 (0.1)	200 (0.5)	0.230
Peripheral vascular disease	2430 (5.8)	62 (6.5)	2368 (5.8)	0.755
Psychiatric disorder	381 (0.9)	4 (0.4)	377 (0.9)	0.165
Pulmonary circulatory disorder	1994 (4.7)	85 (8.9)	1909 (4.6)	0.064
Renal failure	5185 (12.3)	102 (10.6)	5083 (12.3)	0.262
Rheumatoid arthritis and/or collagen vascular disease	1089 (2.6)	27 (2.8)	1062 (2.6)	> 0.999
Valvular heart disease	2570 (6.1)	55 (5.7)	2515 (6.1)	0.476
Weight loss	3803 (9.0)	158 (16.5)	3645 (8.9)	0.002

^1^Comparisons were conducted using the z-test with p-values adjusted using the Bonferroni method

The predictors (aOR; 95% CI; p-value) of APE were coagulopathies (adjusted Odds Ratio [aOR]: 1.57, 95% confidence interval [CI] 1.31–1.89, p < 0.001), Fluid and Electrolyte Disorders (aOR: 2.54, CI 2.13–3.03, p < 0.001), Liver Disease (aOR: 1.37, CI 1.07–1.76, p = 0.013), Obesity (aOR: 1.47, CI 1.19–1.81, p = 0.003), Pulmonary Circulatory Disorder (aOR: 1.72, CI 1.31–2.26, p = 0.001), and Weight Loss (aOR: 1.67, CI: 1.36–2.04, p < 0.001). The results of multivariable logistic regression analysis are given in Table 3.

**Table 3:** Predictors of acute pulmonary edema

Category	Odds ratio (95% CI)*	p value
Age	0.99 (0.98–1.00)	0.090
Gender		
Female	1.0 (0.86–1.16)	> 0.999
Male	Reference	
Selected comorbidities		
Coagulopathy	1.57 (1.31–1.89)	< 0.001
Congestive heart failure	1.15 (0.93–1.42)	0.193
Fluid and electrolyte disorders	2.54 (2.13–3.03)	< 0.001
Hypertension, complicated	1.09 (0.75–1.59)	0.653
Liver disease	1.37 (1.07–1.76)	0.013
Metastatic cancer	1.06 (0.73–1.54)	0.761
Neurologic disorders	1.22 (0.98–1.52)	0.079
Obesity	1.47 (1.19–1.81)	0.003
Pulmonary circulatory disorder	1.72 (1.31–2.26)	0.001
Weight loss	1.67 (1.36–2.04)	< 0.001

^*^Multivariate logistic regression adjusted for age, sex, and selected comorbidities

## Discussion

This retrospective study utilized deidentified data from the 2016–2021 NIS database to identify adult inpatients (≥ 18 years) with a primary diagnosis of non-traumatic SAH, categorized into APE and non-APE groups based on ICD-10 coding, with relevant demographic and clinical variables extracted to identify predictors of APE. A total of 42,141 eligible records were scrutinized, among which 960 (2.3%) exhibited APE. Comorbidities such as coagulopathy, fluid and electrolyte imbalance, liver disease, obesity, pulmonary circulatory disorder, and weight loss were identified as predictors of APE.

The epidemiological parameters of SAH such as the mean age of presentation with female predominance in this study align with findings from previous studies [9]. This is because aneurysms are more common in the elderly due to wear and tear, systemic hypertension, and cumulative exposure to other lifestyle risk factors such as smoking and alcohol consumption [10, 11]. Women, in particular, are more prone as estrogen, the predominant female hormone responsible for vessel wall integrity, can fluctuate during pregnancy and menopause [12].

In our study, the prevalence of APE among patients with non-traumatic SAH was 2.3%. However, the clinical diagnosis of APE in the context of SAH remains inherently challenging. This is compounded by issues such as underreporting, misclassification, and missing data in database-based retrospective studies using ICD-10 codes. For instance, a study focusing on aneurysmal SAH reported that pulmonary edema was clinically identified in only 31% of patients. In contrast, post-mortem examinations revealed a markedly higher prevalence of 78% [13]. This discrepancy highlights the limitations of clinical assessment alone in detecting APE. Furthermore, although our dataset did not permit differentiation of APE subtypes, prior research indicates that a substantial proportion of APE cases following acute brain injury, including SAH, may be attributable to NPE [14]. NPE is a distinct, non-cardiogenic form of APE triggered by acute central nervous system insults yet remains underdiagnosed due to the lack of specific diagnostic criteria and its overlap with other cardiopulmonary conditions. Reported prevalence rates of NPE in SAH vary widely, ranging from 2% to as high as 31% in clinical studies [15], further emphasizing the need for improved recognition and more refined diagnostic tools.

NPE is a complex condition arising from the interplay of the central nervous system (CNS), autonomic nervous system, and cardiovascular/pulmonary systems. CNS injury triggers sympathetic overflow through the ANS, leading to catecholamine release, systemic vasoconstriction, increased blood pressure, and venous return elevating pulmonary capillary hydrostatic pressure [6]. Chemical involvement, particularly with neurotransmitters like NMDA and GABA receptors in specific trigger zones particularly the rostral ventrolateral medulla, area postrema, nuclei of the solitary tract, nuclei of A1 and A5, the medial reticulated nucleus, and the dorsal motor vagus nucleus, adds further complexity. Additionally, raised intracranial pressure secondary to the CNS injury leads to the Cushing triad [increased blood pressure (BP), irregular breathing, and bradycardia] further facilitating pulmonary edema development [6]. Moreover, catecholamine surge can lead to myocardial overload and diastolic dysfunction, contributing to pulmonary edema, while increased pulmonary capillary permeability may result from direct endothelial damage or mechanical responses to elevated hydrostatic pressure [6].

The most prevalent comorbidities observed among patients with SAH in this study were hypertension and fluid and electrolyte imbalance. The latter, which significantly increased the odds of developing APE (aOR 2.54), may reflect the systemic physiological stress induced by acute brain injury, including sympathetic overactivation and hypothalamic–pituitary–adrenal axis dysfunction [16]. These mechanisms can impair sodium and water regulation, leading to volume overload and pulmonary capillary transudation [17]. Additionally, electrolyte imbalances such as hyponatremia and hypokalemia are frequently reported in SAH and may worsen cardiac function, alter vascular tone, and disrupt alveolar-capillary membrane stability [17]. Early correction of sodium and potassium abnormalities, close monitoring of fluid balance, and avoiding aggressive fluid resuscitation are essential clinical steps to reduce pulmonary complications in this subgroup.

Coagulopathy, another independent risk factor (aOR 1.57), may exacerbate APE risk by promoting microvascular dysfunction and increasing capillary permeability. Disseminated intravascular coagulation and platelet dysfunction, both of which are known to occur in acute CNS insults, can compromise the integrity of the pulmonary capillary endothelium, facilitating the leakage of protein-rich fluid into the alveolar spaces [18]. Moreover, anticoagulant use—either pre-existing or initiated during hospitalization—may further predispose to subclinical alveolar hemorrhage and complicate pulmonary status [19]. Early identification of coagulation abnormalities and individualized anticoagulation strategies may help prevent such complications. In addition to this, pulmonary circulatory disorders were also found to significantly increase the odds of APE (aOR 1.72), likely reflecting elevated pulmonary arterial pressures and right heart strain, which can raise hydrostatic pressure in the pulmonary capillary bed. This elevation facilitates fluid extravasation into the alveolar spaces, especially in the setting of compromised endothelial integrity [20]. Pulmonary hypertension, embolism, or other right heart dysfunctions that contribute to pulmonary circulatory disorders can aggravate pulmonary edema in neurologically vulnerable patients [21, 22]. Early recognition of such comorbidities using echocardiography or biomarkers like BNP may aid in triaging and optimizing volume and ventilatory management [23]. Liver disease was also found to be a significant risk factor for APE (aOR 1.37), possibly due to a combination of hypoalbuminemia, fluid overload, systemic inflammation, and coagulopathy resulting from impaired hepatic synthesis of clotting factors, all of which can increase pulmonary capillary leakage and predispose to edema [24].

Obesity, though traditionally under-recognized as a risk factor in neurologic emergencies, was found to increase the odds of APE (aOR 1.47) independently. This may be attributed to several overlapping pathophysiological factors: reduced functional residual capacity, decreased lung compliance, increased airway resistance, and higher circulating blood volume all contribute to impaired pulmonary mechanics [1, 20, 25]. Furthermore, obesity is associated with chronic low-grade inflammation and altered adipokine signaling, which may amplify the pulmonary endothelial response to sympathetic overdrive in SAH [26]. Proactive respiratory support—such as early non-invasive ventilation or positive end-expiratory pressure (PEEP) strategies—and head-of-bed elevation may be particularly beneficial in this population [27]. Weight loss (aOR: 1.67), on the other hand, likely represents a surrogate marker for frailty, sarcopenia, or underlying catabolic states. These conditions may impair cardiovascular reserve and limit compensatory responses to hemodynamic stress, ultimately leading to increased pulmonary artery pressures and pulmonary edema [28]. It is likely that the Hunt–Hess grade ≥ 3 SAH is also a predictor of APE as reported in other studies [29] but this grading was not available in the database.

Incorporating targeted early interventions based on these risk profiles—such as rigorous electrolyte monitoring, individualized fluid and anticoagulation management, respiratory optimization in obese patients, and close cardiopulmonary surveillance in patients with underlying liver or pulmonary circulatory disorders—could significantly improve outcomes. These risk factors, many of which are modifiable or manageable with timely interventions, suggest that a multidisciplinary care approach—integrating neurology, internal medicine, pulmonology, and critical care—may improve outcomes. For instance, early identification and correction of electrolyte disturbances, closer attention to nutritional status, and individualized respiratory surveillance in patients with elevated pulmonary vascular resistance could help prevent clinical deterioration [30]. Moreover, these findings challenge the traditional view that pulmonary complications following SAH are predominantly inevitable sequelae of neurological injury [5], and instead underscore the role of systemic, potentially modifiable risk factors—highlighting the need for tailored preventive protocols. These data may also inform clinical decision-making regarding ICU triage, monitoring intensity, and resource allocation in patients with SAH at higher risk for developing APE [31]. Future clinical guidelines may benefit from incorporating these risk indicators to refine existing management strategies.

The main strength of the present study lies in its large sample size, including representation from all U.S. states, ensuring comprehensive nationwide coverage. However, there are certain limitations to acknowledge. As a retrospective study, it inherently faces challenges related to biases such as underreporting, misclassification, and missing data [32]. Further, as the NIS is an administrative dataset, the diagnosis of APE was recorded based on physician coding using ICD-10 codes at discharge. Unfortunately, more clinical validation (e.g., imaging or laboratory data) is unavailable in this data set. Administrative databases typically lack detailed clinical information such as treatment regimens, treatment-related complications (e.g., need for mechanical ventilation, vasospasm, or delayed cerebral ischemia), patient functional status, and the underlying cause of hemorrhage. Crucially, they also do not capture established global severity indices such as the Hunt and Hess scale, the Modified Fisher Score, the World Federation of Neurosurgical Societies (WFNS) scale, the Acute Physiology and Chronic Health Evaluation II (APACHE II) score, or the Sequential Organ Failure Assessment (SOFA) score—limiting the ability to fully adjust for acute illness severity, which is known to influence outcomes in SAH. While comorbidity indices like Elixhauser partially reflect baseline health status, they are not a substitute for physiological scoring systems and may not capture real-time clinical deterioration. In addition, NPE could not be analyzed as a distinct entity due to lack of corresponding variables in the dataset. Although a DAG-based framework was used to minimize selection and modeling bias, the possibility of residual confounding from unmeasured variables remains. These limitations may be better addressed through prospective studies enriched with real-time physiological data, which could further validate the findings of the present study.

To summarize, the incidence of APE over a 6-year period (2016–2021), based on NIS data, was 2.3% among patients with non-traumatic SAH. Coagulopathy, fluid and electrolyte imbalance, liver disease, obesity, pulmonary circulatory disorders, and weight loss were identified as significant risk factors. These findings highlight the importance of early identification and proactive management of high-risk patients to mitigate the risk and impact of APE. Given the inherent limitations of administrative data in distinguishing clinical subtypes such as neurogenic versus cardiogenic pulmonary edema, and lack of real-time clinical and physiologic data, prospective studies with detailed clinical and imaging data are warranted.

## Supplementary Information

Supplementary Material 1. Supplementary Table 1: International Classification of Diseases, 10th Revision, (ICD – 10) Clinical Modification (CM) codes used for comorbidities. Supplementary Table 2: Causal Framework-Based Stratification of Comorbidities Associated with Acute Pulmonary Edema in Sub-Arachnoid Haemorrhage. Supplementary Figure 1: Simplified Directed Acyclic Graph Depicting Presumed Risk Factors for Acute Pulmonary Edema Following Sub-Arachnoid Haemorrhage.

## Data Availability

The dataset used in this study is publicly available from the Healthcare Cost and Utilization Project (HCUP) National Inpatient Sample (NIS) at https://www.hcup-us.ahrq.gov/nisoverview.jsp

## Abbreviations

AHRQ: Agency for Healthcare Research and Quality
aOR: Adjusted Odds Ratio
APACHE: Acute Physiology and Chronic Health Evaluation
APE: Acute pulmonary edema
BNP: B-type natriuretic peptide
CI: Confidence interval
HCUP: Healthcare Cost and Utilization Project
HPA: Hypothalamic–Pituitary–Adrenal
ICD-10-CM: International Classification of Diseases, 10th Revision, Clinical Modification
NIS: National Inpatient Sample
NPE: Neurogenic Pulmonary Edema
PEEP: Positive end-expiratory pressure
SAH: Subarachnoid hemorrhage
SOFA: Sequential Organ Failure Assessment
