# Supplementary material for: Acute pulmonary edema after subarachnoid hemorrhage: risk factors and comorbidities—an analysis of a nationwide database from the United States
Source: J Intensive Care. 2025 May 13;13:25. doi: 10.1186/s40560-025-00796-w (PMC12070654; doi:10.1186/s40560-025-00796-w)

**Supplementary Table – 1: International Classification of Diseases, 10th Revision, (ICD – 10) Clinical Modification (CM) codes used for comorbidities**

| Category | ICD-10 CM Codes |
| --- | --- |
| Acquired Immune Deficiency Syndrome (AIDS) | B20–B24 |
| Alcohol abuse | F10 |
| Blood Loss Anemia | D62 |
| Cardiac Arrhythmias | I47–I49 |
| Chronic deficiency anemia | D50.0–D50.9 |
| Chronic lung disease | J40–J47 |
| Coagulopathy | D65–D69 |
| Congestive heart failure | I50 |
| Depression | F32–F33 |
| Diabetes mellitus, uncomplicated | E11.9 |
| Diabetes mellitus, complicated | E11.0–E11.8 |
| Drug abuse | F11–F19 |
| Fluid and electrolyte disorder | E86–E87 |
| Hypertension, uncomplicated | I10 |
| Hypertension, complicated | I11–I15 |
| Hypothyroidism | E03 |
| Liver disease | K70–K77 |
| Neurologic disorders | G00–G99 (excluding G80 - G83) |
| Obesity | E66 |
| Solid tumors without metastasis | C00–C80 (excluding C77–C79) |
| Metastatic cancer | C77–C79 |
| Lymphoma | C81–C85 |
| Paralysis | G80–G83 |
| Peptic ulcer disease excluding bleeding | K25–K28 (excluding codes with .0 or .1) |
| Peripheral vascular disease | I73 |
| Psychiatric disorder | F20–F29 |
| Pulmonary circulatory disorder | I26–I28 |
| Renal failure | N17–N19 |
| Rheumatoid arthritis and/or collagen vascular disease | M05–M06 |
| Valvular heart disease | I34–I39 |
| Weight loss | R63.4 |

**Supplementary Table – 2: Causal Framework-Based Stratification of Comorbidities Associated with Acute Pulmonary Edema in Sub-Arachnoid Haemorrhage**

| Comorbidity | Rationale |
| --- | --- |
| **✅ Likely causal risk factor** | |
| Coagulopathy | Increases the risk of capillary leak or pulmonary hemorrhage. |
| Congestive Heart Failure | Direct elevation of hydrostatic pressure; strongly linked to pulmonary edema. |
| Fluid and Electrolyte Disorder | Causes fluid shifts, volume overload, and increases risk of pulmonary edema. |
| Hypertension, Complicated | May reflect target-organ damage like LVH or subclinical heart failure, which increases acute pulmonary edema risk |
| Liver Disease | Hypoalbuminemia and volume overload contribute to pulmonary edema. |
| Metastatic Cancer | Systemic inflammation and lymphatic obstruction increase risk. |
| Neurologic Disorders | Conditions like Sub-arachnoid Haemorrhage can cause neurogenic pulmonary edema. |
| Renal Failure | Reduces fluid clearance; volume overload is a common cause of acute pulmonary edema. |
| Obesity | Linked to diastolic dysfunction, higher thoracic pressure, and volume overload. |
| Pulmonary Circulatory Disorder | Pulmonary hypertension or embolism directly cause capillary pressure elevation. |
| Weight Loss | Represents frailty and vulnerability to fluid shifts in critical illness. |
| **⚠️ Conditionally relevant** | |
| Cardiac Arrhythmias | Can destabilize hemodynamics, especially in decompensated patients. |
| Hypertension (Complicated) | May contribute to left ventricular dysfunction or co-exist with other comorbidities like renal failure |
| Diabetes Mellitus, Complicated | May indirectly increase acute pulmonary edema risk through nephropathy or vascular changes. |
| Valvular Heart Disease | May lead to pulmonary edema if severe (e.g., mitral regurgitation). |
| **❌ Unlikely or non-causal** | |
| Hypertension, Uncomplicated | No end-organ damage; may act as a collider in observational models. |
| Hypothyroidism | Rare cause of edema unless severe; often subclinical. |
| Solid Tumors without Metastasis | Rarely cause acute pulmonary edema; may introduce collider bias. |
| Psychiatric Disorders | No pathophysiologic link to fluid overload. |
| Depression | No biologic basis for direct causation of acute pulmonary edema. |
| AIDS | May increase infection risk, but not directly linked to acute pulmonary edema. |
| Drug Abuse / Alcohol Abuse | Too broad a category in datasets; not reliably linked to acute pulmonary edema. |
| Peripheral Vascular Disease | Atherosclerotic; not a driver of pulmonary fluid accumulation. |
| Peptic Ulcer Disease | No plausible link to acute pulmonary edema. |
| Blood Loss Anemia / Chronic Anemia | Impacts oxygenation, not fluid dynamics. |
| Lymphoma | Unlikely to cause acute pulmonary edema unless mediastinal mass effect. |
| Paralysis | Does not cause acute pulmonary edema; increased risk for other complications. |
| Rheumatoid Arthritis / Collagen Vascular Disease | May affect lungs rarely; not typically causing acute pulmonary edema. |

**Supplementary Figure – 1: Simplified Directed Acyclic Graph Depicting Presumed Risk Factors for Acute Pulmonary Edema Following Sub-Arachnoid Haemorrhage**


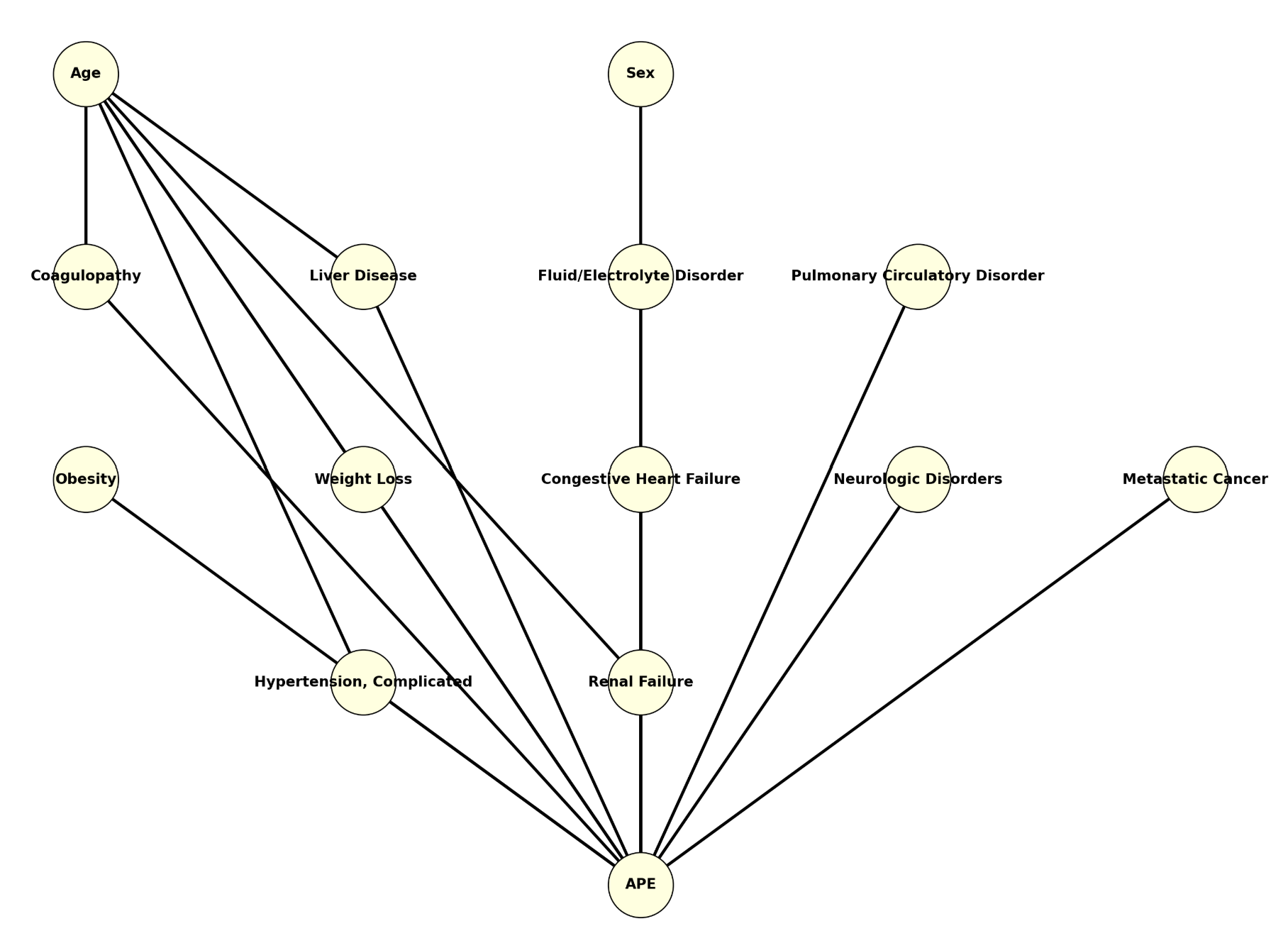

Supplement: Supplementary file 1 — Supplementary Material 1. Supplementary Table 1: International Classification of Diseases, 10th Revision, (ICD – 10) Clinical Modification (CM) codes used for comorbidities. Supplementary Table 2: Causal Framework-Based Stratification of Comorbidities Associated with Acute Pulmonary Edema in Sub-Arachnoid Haemorrhage. Supplementary Figure 1: Simplified Directed Acyclic Graph Depicting Presumed Risk Factors for Acute Pulmonary Edema Following Sub-Arachnoid Haemorrhage. [file 40560_2025_796_MOESM1_ESM.docx]
